# C-reactive protein and white matter microstructural changes in COVID-19 patients with encephalopathy

**DOI:** 10.1007/s00702-021-02429-6

**Published:** 2021-10-28

**Authors:** Alexandra Rhally, Alessandra Griffa, Stéphane Kremer, Marjolaine Uginet, Gautier Breville, Patrick Stancu, Frédéric Assal, Patrice H. Lalive, Karl-Olof Lövblad, Gilles Allali

**Affiliations:** 1 Faculty of Medicine, University of Geneva, Geneva, Switzerland; 2 Department of Clinical Neurosciences, Division of Neurology, Geneva University Hospitals and Faculty of Medicine, University of Geneva, Geneva, Switzerland; 3 Institute of Bioengineering, Center of Neuroprosthetics, Ecole Polytechnique Fédérale de Lausanne (EPFL), Campus Biotech-H4 3 232.080 (H4 building), Chemin des Mines 9, Case postale 60, CH-1211 Geneva, Switzerland; 4 Service d’imagerie 2, Hôpitaux Universitaires de Strasbourg, Strasbourg, France; 5 Engineering Science, Computer Science and Imaging Laboratory (ICube), Integrative Multimodal Imaging in Healthcare, UMR 7357, University of Strasbourg-CNRS, Strasbourg, France; 6 Diagnostic Department, Division of Laboratory Medicine, Geneva University Hospitals, Geneva, Switzerland; 7 Department of Pathology and Immunology, Faculty of Medicine, University of Geneva, Geneva, Switzerland; 8 Division of Neuroradiology, Geneva University Hospitals and University of Geneva, Geneva, Switzerland; 9 Division of Cognitive and Motor Aging, Department of Neurology, Albert Einstein College of Medicine, Yeshiva University, Bronx, NY USA

**Keywords:** COVID-19, DWI, CRP, Encephalopathy, Inflammation, White matter

## Abstract

Encephalopathy is a neurological complication of COVID-19. The objective of this exploratory study is to investigate the link between systemic inflammation and brain microstructural changes (measured by diffusion-weighted imaging) in patients with COVID-19 encephalopathy. 20 patients with COVID-19 encephalopathy (age: 67.3 $\pm$ 10.0 years; 90% men) hospitalized in the Geneva University Hospitals for a SARS-CoV-2 infection between March and May 2020 were included in this retrospective cohort study. COVID-19 encephalopathy was diagnosed following a comprehensive neurobiological evaluation, excluding common causes of delirium, such as hypoxemic or metabolic encephalopathy. We investigated the correlation between systemic inflammation (measured by systemic C-reactive protein (CRP)) and brain microstructural changes in radiologically normal white matter (measured by apparent diffusion coefficient (ADC)) in nine spatially widespread regions of the white matter previously associated with delirium. Systemic inflammation (CRP = 60.8 ± 50.0 mg/L) was positively correlated with ADC values in the anterior corona radiata (*p* = 0.0089), genu of the corpus callosum (*p* = 0.0064) and external capsule (*p* = 0.0086) after adjusting for patients’ age. No statistically significant association between CRP and ADC was found in the other six white matter regions. Our findings indicate high risk of white matter abnormalities in COVID-19 encephalopathy patients with high peripheral inflammatory markers, suggesting aggressive imaging monitoring may be warranted in these patients. Future studies should clarify a possible specificity of the spatial patterns of CRP–white matter microstructure association in COVID-19 encephalopathy patients and disentangle the role of individual cytokines on brain inflammatory mechanisms.

## Introduction

Coronavirus disease 2019 (COVID-19) induced by severe acute respiratory syndrome coronavirus-2 (SARS-CoV-2) infection has been associated with neurological complications, such as idiopathic encephalopathy (Helms et al. 2020a, b). The clinical spectrum of the COVID-19 encephalopathy is broad, ranging from delirium to coma. An inflammatory hypothesis has been suggested for the neuropathogenesis of this encephalopathy (Teuwen et al. 2020; Najjar et al. 2020; Fotuhi et al. 2020). Although a possible disruption of brain microstructural integrity has been demonstrated in COVID-19 patients (Lu et al. 2020; Newcombe et al. 2020), the relationship between systemic inflammation and microstructural brain changes has been poorly investigated in patients with COVID-19 encephalopathy.

The apparent diffusion coefficient (ADC), a measure derived from diffusion-weighted imaging (DWI) quantifying the local amount of water molecule diffusion, has been largely used to assess white matter microstructure (Alexander et al. 2007). White matter changes in the corpus callosum, thalamocortical, limbic, and cerebellar circuits have been associated with delirium (Nitchingham et al. 2018). To investigate the link between systemic inflammation and brain microstructural changes in COVID-19 encephalopathy patients, we studied the association between C-reactive protein (CRP) levels and ADC in nine white matter regions previously associated with delirium.

## Materials and methods

### Standard protocol approvals, registrations, and patients consents

The study was approved by the institutional review board of Geneva University Hospitals (protocol #2020-01206—approved May 25, 2020) and has been performed in accordance with the ethical standards laid down in the 1964 Declaration of Helsinki and its later amendments.

De-identified data will be made available to qualified investigators upon written request to the last author.

### Patient cohort

From March to May 2020, 41 patients had been diagnosed with COVID-19 encephalopathy out of 707 COVID-19 patients hospitalized in Geneva University Hospitals. SARS-CoV-2 infection was confirmed by a positive SARS-CoV-2 reverse transcription polymerase chain reaction (RT-PCR) assay from a nasopharyngeal swab at the time of hospitalization. Diagnosis of COVID-19 encephalopathy was defined by a rapidly developing (less than 4 weeks) brain pathological process leading to delirium, decreased level of consciousness or coma (Helms et al. 2020b), excluding classical etiologies for delirium such as hypoxemia, electrolyte disturbances, infection, drug or alcohol toxicity, metabolic disorders, low perfusion state or acute central nervous system conditions such as meningitis. The severity of the encephalopathy was assessed with the Richmond Agitation–Sedation Scale (RASS) (a patient with an RASS score of − 5 being unarousable (unresponsive to voice and physical stimulation) and a patient with an RASS score of + 4 being combative (attempts to stand or get out of bed; danger to self or staff)), and with the duration of encephalopathy. Clinical data, CSF RT-PCR for SARS-CoV-2, CSF white blood cell count (WBC), CSF/serum quotient of albumin (QAlb), and blood CRP levels at the time of brain magnetic resonance imaging (MRI) were retrospectively collected from the patients’ records. Patients with incomplete MRI data, image processing failure or more than 48-h delay between CRP assessment and MRI were excluded. Twenty patients were included in the final analyses (67.3 ± 10.0 years; 18 men), of which 11 had CSF analysis (Table1).

**Table 1:** Clinical characteristics

	All participants
No. of participants	20
Age (years), mean ± SD (range)	67.3 $\pm$ 10.0 (46.6 to 80.03)
Sex, men *N* (%)	18 (90%)
BMI^a^ (kg/m^2^), mean ± SD (range)	27.3 $\pm 2.8$ (21.9 to 31.2)
Stroke, *N* (%)	2 (10%)
CRP (mg/L), mean ± SD (range)	60.8 $\pm$ 50.0 (8.0 to 153.0)
Severity of encephalopathy	
RASS^b^, median (range)	− 1 (− 4 to − 2)
COVID-19 encephalopathy duration (days), mean ± SD (range)	13.6 $\pm$ 8.6 (4.0 to 32.0)
CSF analyses	
White blood cell count, mean ± SD (range)	2.09 ± 2.47 (0.00 to 9.00)
CSF/serum quotient of albumin, mean ± SD (range)	12.37 ± 7.49 (4.56 to 28.58)

^a^Body mass index

^b^Richmond Agitation–Sedation Scale

### Brain imaging

All 20 patients underwent an MRI scan at Geneva University Hospitals on a 1.5 Tesla Philips Ingenia system equipped with a multi-channel head coil. The MRI scan included a structural and a diffusion-weighted sequence with the following parameters: 2D gadolinium-enhanced T1-weighted gradient echo sequence, transverse acquisition, in-plane resolution 0.4 × 0.4 mm^2^, acquisition matrix = 512 × 512 pixels; slice thickness = 5 mm, spacing between slices = 5.3 mm, number of slices = 30, number of averages = 2, flip angle = 80°, TE = 2.4 ms, TR = 259 ms; 2D diffusion-weighted spin echo sequence, transverse acquisition, in-plane resolution = 1.3 × 1.3 mm^2^, acquisition matrix = 176 × 176 pixels, slice thickness = 4 mm, spacing between slices = 4.4 mm, number of slices = 34, number of averages = 2, flip angle = 90°, TE = 75 ms, TR = 4061 ms, diffusion *b*-value = 1000 s/mm^2^.

### Image analyses

The average DWI b1000 images were inspected by two certified neuroradiologists to assess the presence of stroke. Acute stroke lesions were semi-automatically segmented on the b1000 images using the Clusterize SPM12 toolbox (Clas et al. 2012). The T1-weighted volumes were segmented into brain tissue compartments and co-registered to the diffusion volumes using SPM12 (Penny et al. 2006). Regions of interest in the white matter were extracted using an automatic segmentation based on the ICBM-DTI-81 white matter atlas (Mori et al. 2008). In particular, the atlas was warped to individual T1-weighted images to identify white matter regions in native space. Voxel not belonging to the white matter according to the SPM12 segmentation were excluded from the regions of interest (threshold on individual white matter tissue probability maps: 0.8). In addition, to ensure that results were not driven by the presence of stroke, all analyses were repeated excluding the patients with acute stroke (*N* = 2). Based on previous literature on delirium (Nitchingham et al. 2018), nine bilateral white matter regions were retained for further analyses, namely, the genu and splenium of the corpus callosum, the posterior thalamic radiation, the anterior corona radiata, the cingulum, the uncinate fasciculus, the hippocampal white matter, the external capsule and the middle cerebellar peduncle. Of note, the uncinate fasciculus is part of a dense network of white matter fibers that interconnect different structures of the olfactory sensory pathway, and the investigation of this region may further elucidate the possible implication of the olfactory route regarding neuro-invasion (Lopez-Elizalde et al. 2018). An average ADC value was computed for each white matter region (average between left and right hemispheres), excluding voxels belonging to acute stroke lesions. In addition, analyses were repeated considering regions belonging to the left and right hemispheres separately to assess possible lateralization effects. Image analyses were performed with MATLABv9.7 and SPM12.

### Statistical analyses

Linear regression analyses were used to assess the associations between the average ADC in nine white matter regions and CRP levels. Age was added as covariate. False discovery rate was controlled at FDR = 0.05 to correct for multiple comparisons (Benjamini and Hochberg 1995). Spearman’s rank correlation was used in exploratory analyses to assess possible relationships between CRP levels or ADC in different brain regions, and severity of encephalopathy as quantified with the RASS score and the duration of encephalopathy.

## Results

The clinical characteristics of the COVID-19 encephalopathy patients are presented in Table 1. The majority of patients were men and their CRP levels ranged from 8.0 to 153.0 mg/L. The RASS scores ranged from − 4 to − 2, with a median of − 1, indicating that the majority of patients suffered a mild form of encephalopathy. Duration of encephalopathy ranged from 4 to 32 days, with a mean duration of 13.6 ± 8.6 days. 11 out of 20 patients had a CSF analysis: the mean CSF white blood cell count was 2.09 ± 2.47 leukocytes/mm^3^, whereas CSF/serum quotient of albumin (QAlb—measured in 8 out of 20 patients) was increased in 75% (mean QAlb = 12.37 ± 7.49). CSF RT-PCR for SARS-CoV-2 was negative for all patients (measured in 10 out of 20 patients).

**Fig. 1:**
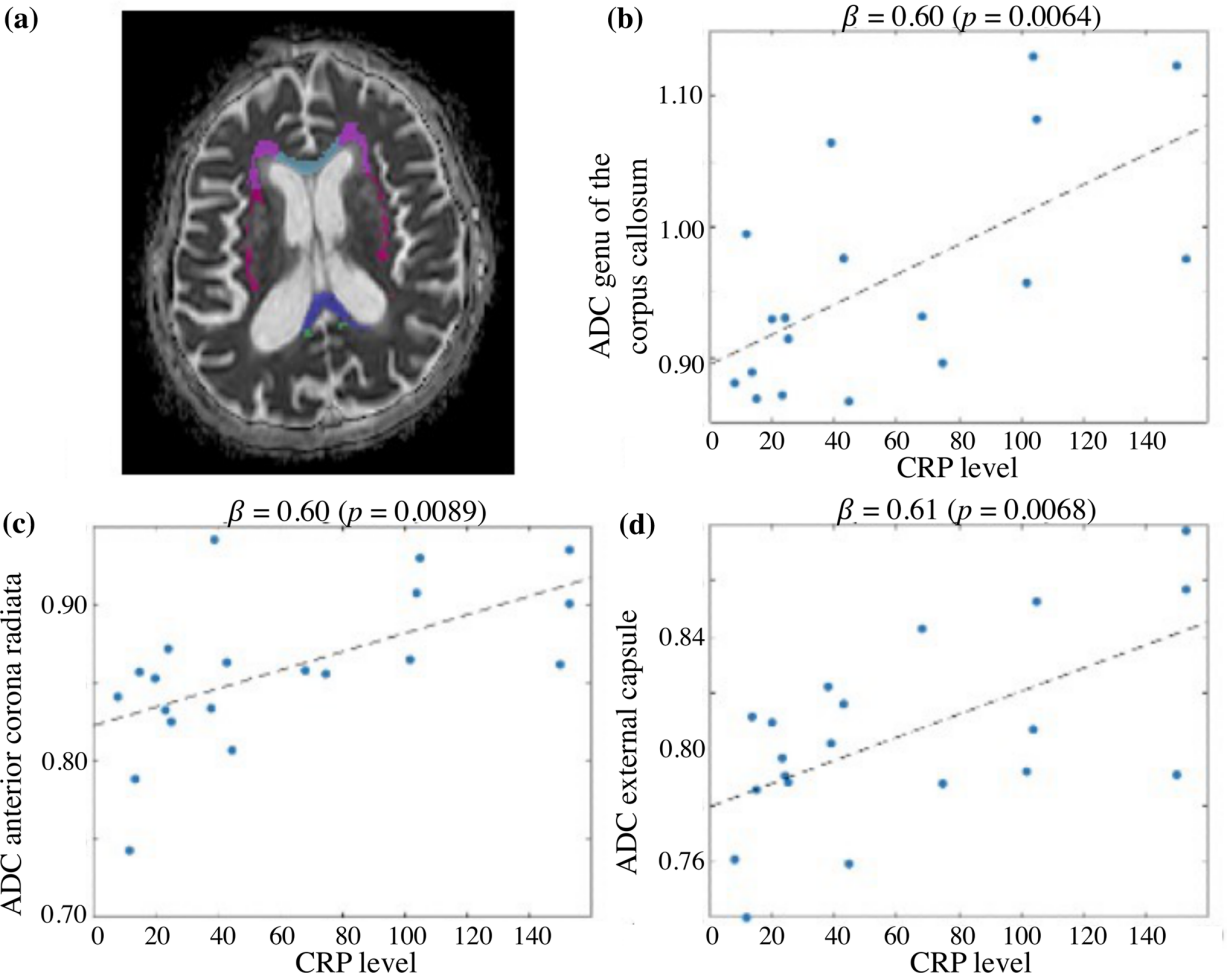
Association between systemic inflammation and white matter microstructure. **a** Example of ADC map. Colored regions represent the genu of the corpus callosum (light blue), anterior corona radiata (lilac), external capsule (violet), splenium of the corpus callosum (dark blue) and cingulum (green). **b**–**d** Scatter plots representing the association between CRP levels and average ADC in the genu of the corpus callosum, anterior corona radiata and external capsule. Standardized beta coefficients and *p* values are reported. Each dot in the plots represents a patient; the dashed lines represent the best linear fits

Regression analyses with the average ADC in a white matter region as predicted variable, the CRP level as predictor variable, and the age as covariate, showed that higher CRP levels were significantly associated with increased ADC in the genu of the corpus callosum (*β* = 0.60, *t*(13) = 3.16, *p* = 0.0064), anterior corona radiata (*β* = 0.60, *t*(15) = 2.95, *p* = 0.0089), and external capsule (*β* = 0.61, *t*(15) = 2.97, *p* = 0.0068) (Fig. 1). These associations survived multiple comparison correction. No CRP–ADC association was found in the other six white matter regions. Repeating the analyses only on patients with no acute stroke (*N* = 18) did not change results (genu of the corpus callosum: *β* = 0.49, *t*(11) = 2.58, *p* = 0.023; anterior corona radiata: *β* = 0.69, *t*(13) = 3.44, *p* = 0.0036; external capsule: *β* = 0.66, *t*(13) = 3.11, *p* = 0.0072; associations between anterior corona radiata and external capsule ADC, and CRP levels survived FDR correction). No hemispheric lateralization of CRP–ADC associations was found when performing the analyses considering the left and right hemisphere separately (linear associations between CRP and ADC were significant with uncorrected *p* value < 0.05 for both left and right anterior corona radiata (*β* = 0.58, *t*(15) = 2.86, *p* = 0.011; *β* = 0.60, *t*(15) = 2.75, *p* = 0.014; respectively), and left and right external capsule (*β* = 0.51, *t*(15) = 2.32, *p* = 0.033; *β* = 0.62, *t*(15) = 3.11, *p* = 0.0063; respectively)). Finally, there were no significant associations between CRP levels or ADC in different brain regions, and severity of encephalopathy as measured with the RASS score or the encephalopathy duration.

## Discussion

In this preliminary study, we found that in COVID-19 encephalopathy patients, systemic inflammation (measured by CRP levels at the time of brain MRI) is associated with white matter microstructural changes (measured by ADC) in the anterior corona radiata, genu of the corpus callosum and external capsule. This association was specific to frontal (genu of the corpus callosum, anterior corona radiata) and cholinergic (external capsule) projections—no association was found in other limbic, thalamocortical, cerebellar, or olfactory regions. These findings are consistent with the cognitive profile of COVID-19 patients with encephalopathy, mainly presenting a fronto-subcortical syndrome (Beaud et al. 2020), and with the pathogenesis of delirium that can be triggered by acute inflammation (Wilson et al. 2020). Further analyses including comprehensive neuropsychological assessments are needed to understand whether neuropathological mechanisms related to systemic inflammation may impact frontal lobe functions.

The presence of an association between systemic inflammation and white matter microstructure in the corpus callosum is coherent with studies describing cytokine-induced injury in the corpus callosum in critical illnesses, such as acute respiratory distress syndrome and COVID-19 (Cannac et al. 2020), and a more frequent occurrence of white matter neurological events in this very same region in COVID-19 patients (Parsons et al. 2020). Furthermore, the increased quotient albumin reported in the majority of our patients with available CSF analyses suggests a blood–brain barrier disruption associated with this systematic inflammation, which may explain the observed white matter microstructural changes. However, fronto-cholinergic white matter microstructural changes may be particularly prevalent in COVID-19 encephalopathy, as research on other pathogen-driven encephalopathies found different brain regions. For example, acute necrotizing encephalopathy has been associated with restricted diffusion imaging in the centrum semiovale, thalami, pontine tegmentum, cerebellar vermis, and deep cerebellar hemispheres (Bailey 2020). In the case of influenza-associated acute encephalopathy, restricted diffusion has been found in widespread white matter regions (Tsuchiya et al. 2000) and in the hippocampus (Koll et al. 2020). Moreover, the CRP–ADC associations in the anterior corona radiata and the external capsule highlighted by our analyses are consistent with previous research conducted on COVID-19 patients (Lu et al. 2020). However, we did not find any hemispheric lateralization of association between CRP and ADC changes in our analyses. Associations between CRP levels and white matter microstructure should be further investigated in future studies on larger cohorts, to explore the possible spatial specificity of effects to different virus-related encephalopathies.

White matter microstructure was assessed with the ADC, a measure of water molecule diffusion in brain tissues. ADC changes are considered to unspecifically reflect multiple microstructural mechanisms including axonal degeneration, loss of microstructural integrity as well as local inflammation (Alexander et al. 2007). Our findings suggest that systemic inflammation may support one or more of these neuropathological mechanisms resulting in ADC alteration. Inclusion of more advanced diffusion MRI sequences beyond DWI and diffusion tensor imaging remains mandatory for a thorough characterization of the microstructural mechanisms relating to inflammation, which, however, is impractical in critical clinical settings (Novikov et al. 2018; Alexander et al. 2017).

Our exploratory correlation analyses between systemic inflammation or white matter microstructure and the severity of encephalopathy, as recorded with the RASS score and the duration of encephalopathy, did not show any significant association between these variables. Various alternate mechanisms may contribute to the severity of encephalopathy, such as medication or comorbidities. Furthermore, delirium is highly fluctuating, and a RASS score recorded at a single time point may not fully represent the patients’ clinical condition. The use of more sensitive measures of encephalopathy may help to better address the relationship between biological markers and clinical presentation.

Various mechanisms have been suggested to explain COVID-19 encephalopathy, involving a direct viral invasion through the olfactory retrograde route or an inflammatory mechanism during the so-called cytokine storm inducing blood–brain barrier (BBB) alterations (Iadecola et al. 2020). Several findings support the latter hypothesis, such as an increased CSF/serum quotient albumin in COVID-19 encephalopathy patients suggestive of a leaky BBB, and SARS-CoV-2-associated cytokines (interleukin-6, IL-1β, tumor necrosis factor and IL-17, among others) which are known to disrupt the BBB (Iadecola et al. 2020). Moreover, COVID-19 patients with encephalopathy have negative PCR SARS-CoV-2 in the CSF, suggesting that encephalopathy is not directly related to viral infection but to the inflammatory response. Post-mortem biopsy evidence of direct viral involvement in the brain or olfactory nerve is limited although this might be due to technical limitations (Mukerji and Solomon 2021; Butowt et al. 2021). On this line, we did not find any significant associations between systemic inflammation and white matter microstructure of the uncinate fasciculus, a fiber bundle that is part of the olfactory pathways (Lopez-Elizalde et al. 2018). The current findings showing an association between systemic inflammation and white matter microstructure in frontal and cholinergic projections support the inflammatory hypothesis.

Associations between serum lactate dehydrogenase (LDH) levels and DTI metrics have been observed in COVID-19 patients in a 3-month follow-up study (Lu et al. 2020), which is in line with our conclusion regarding the associations between microstructural changes in the white matter and CRP. Elevated LDH is a consequence of tissue damage (Augoff et al. 2015) and has been observed in various pathologies, such as encephalitis, ischemic stroke or head injuries (Valvona et al. 2016). Moreover, LDH levels have also been correlated with acute encephalopathy and could be related to prognosis as a significant difference in serum LDH levels was observed between pediatrics patients with and without developmental regression following acute encephalopathy (Motojima et al. 2016). Future research should evaluate if this association between serum LDH levels and DTI metrics in COVID-19 patients is related to neurological complications, such as encephalopathy, and if it is the case, whether it is a good predictor of outcome regarding neurological sequelae.

The association between systemic inflammation and white matter microstructure highlighted by our results is a snapshot of COVID-19 patients at the time of clinical presentation of encephalopathy. Regarding the long-term consequences of COVID-19 encephalopathy, recent studies suggest that this encephalopathy could have long-term effects on brain and cognition. First, microstructural changes in the white matter have been reported not only at the time of infection, but also up to 3 months after hospital discharge (Lu et al. 2020; Raman et al. 2021). A longitudinal PET study showed brain hypometabolism in prefrontal, insular, and middle temporal regions, with abnormalities persisting at 6 months follow-up (Kas et al. 2021). These findings indicate that brain integrity remains affected even months after SARS-CoV-2 infection. Persistent neuroinflammation has also been proposed as a possible explanation regarding long-term MRI changes seen in COVID-19 patients (Goldberg et al. 2020), and follow-up inflammatory markers correlate with severity of COVID-19 at admission (Raman et al. 2021). Second, impaired cognitive performances have been observed in COVID-19 patients compared to a control group in a follow-up study (Raman et al. 2021). Moreover, the incidence of neurological or psychiatric diagnoses—including dementia—following COVID-19 has been estimated at 34%, higher than in a control group of patients who had influenza or other respiratory tract infection, and it increases to 62% for patients who presented a COVID-19 encephalopathy (Taquet et al. 2021). COVID-19, especially when complicated by COVID-19 encephalopathy, may therefore reveal, amplify, or accelerate subjacent neurological or psychiatric diagnosis and may lead to long-term clinical sequelae.

Although this highly selected sample of COVID-19 encephalopathy patients with brain imaging during acute presentation and CRP assessment at the time of imaging are major strengths of this exploratory study, the retrospective nature of this study has limitations. First, only blood C-reactive protein levels were available for these patients at the time of MRI, which limits our ability to draw conclusions using other inflammatory markers than CRP that might be more specific to central nervous system inflammation. Future studies should include a cytokine profile to disentangle the pathogenesis of this encephalopathy and other inflammatory markers, such as fibrinogen and IL-6, as CSF cytokine alterations have been highlighted in COVID-19 encephalopathy patients (Benameur et al. 2020). Second, we did not compare COVID-19 encephalopathy patients with another group of patients with virus-related encephalopathy (e.g., influenza), or with a group of Intensive Care Unit patients (independently from the type of pathology). Therefore, we cannot conclude that our results are specific to SARS-CoV-2 infection. Future studies should compare the strength of association between CRP and ADC values between different pathogen-driven encephalopathy groups to be able to conclude if these associations are specific to certain pathogens or to pathogen-related encephalopathies in general. Indeed, CRP levels have been correlated with white matter lesions in multiple pathologies not related to COVID-19, such as cerebral small vessel disease, neurodegenerative processes and obesity (Mitaki et al. 2015; Eagen et al. 2012; Lampe et al. 2018), which may occur as comorbidities in COVID-19 encephalopathy patients. The limited sample size of this preliminary study did not allow to control for confounders and comorbidities that could affect both the white matter integrity and the CRP values. We believe that a robust investigation in this direction will need to include larger samples and multiple patient groups to disentangle the role of specific pathophysiological mechanisms, inflammation markers and brain structure. In particular, it will be of interest to understand whether the fronto-cholinergic spatial patterns of association between white matter integrity and systemic inflammation are specific to COVID-19 encephalopathy. Finally, in this study white matter microstructure was assessed with the ADC parameter only. Future studies including more advanced diffusion MRI sequences as well as T2/FLAIR and susceptibility weighted imaging MRI contrasts among others (O’Donovan et al. 2021; Rahmanzade et al. 2020) are needed for a thorough characterization of white matter signal abnormalities and cerebrovascular factors to further explore the inflammatory damage and its relation to viral infections.

## Conclusions

In conclusion, our findings demonstrate an association between a peripheral inflammatory marker and white matter microstructural changes in frontal and cholinergic projections, supporting an inflammatory pathogenesis to COVID-19 encephalopathy. These results may provide a rationale to support the use of anti-inflammatory drugs for treating this encephalopathy.

## Data Availability

De-identified data will be made available to qualified investigators upon written request to the last author.
